# Successful Treatment of Pulmonary Scedosporiosis With Voriconazole and Lobectomy in a Patient With Interstitial Pneumonia

**DOI:** 10.1002/rcr2.70181

**Published:** 2025-04-21

**Authors:** Takahiro Masuda, Ayako Yoshiyama, Sayaka Fujimoto, Yuya Furukawa, Hiroko Jinno, Soichiro Yoshimura, Masatsugu Yamamoto, Kimihide Tada

**Affiliations:** ^1^ Department of Respiratory Medicine Nishi‐Kobe Medical Center Kobe Japan

**Keywords:** interstitial pneumonia, *Scedosporium apiospermum*, surgical intervention, voriconazole

## Abstract

This is a rare case of fungal infection, with *Scedosporium apiospermum*, which was correctly diagnosed and treated successfully with anti‐fungal therapy and surgical section. The prompt diagnosis and treatment strategy are both important for successful patient outcomes.

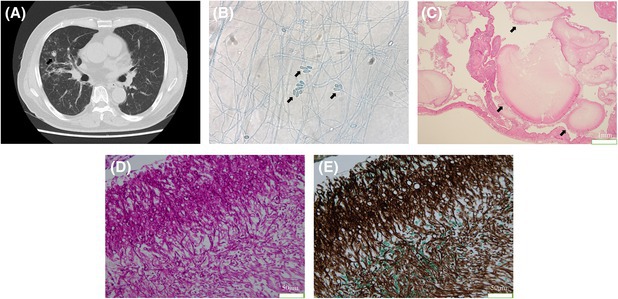

1

The patient was a 78‐year‐old man diagnosed with interstitial pneumonia who had remained stable long‐term with steroid treatment. Routine follow‐up chest CT revealed multiple new nodular lesions in the right middle lobe (Figure 1A). Bronchoscopy was performed, and *Scedosporium apiospermum* was identified in the bronchoalveolar fluid (Figure 1B). This led to a diagnosis of pulmonary scedosporiosis. Accordingly, treatment with voriconazole was initiated. He received treatment for 2 months; however, there was no noticeable improvement in his condition. Due to the localised nature of the disease, a thoracoscopic right middle lobectomy was performed (Figure 1C–E). Postoperative recovery was smooth, and he received voriconazole for 6 months, with no signs of the infection returning. *S. apiospermum* is a filamentous saprophytic fungus that causes various infections in humans, especially in individuals with weakened immune systems [1]. Voriconazole is widely recognised as the preferred treatment for *S*. *apiospermum* infections. It has been proven to be more effective than other antifungal agents and is typically used as a standalone therapy. In specific cases such as localised infections or abscesses, surgical debridement or resection may be needed along with antifungal therapy [2]. This approach may be particularly beneficial in patients with limited response to antifungal therapy alone.

**FIGURE 1:**
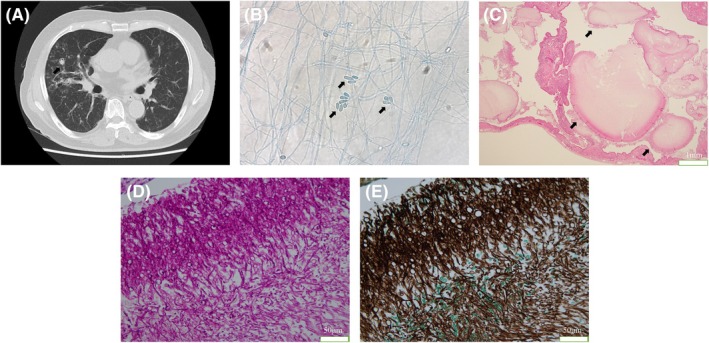
(A) Computed tomography scan showing multiple nodular lesions (arrow) in the right middle lobe. (B) Saprophytic filamentous fungus in the bronchoalveolar fluid of the right middle lobe (arrow) (lactophenol cotton blue staining; ×400). (C–E) A VATS specimen was obtained from the right middle lobe. (C) Large fungal masses within pulmonary cysts (arrow) (haematoxylin and eosin staining, ×100). (D and E) Saprophytic filamentous fungus (Periodic acid‐Schiff staining, D: ×400) (Grocott staining, E: ×400).

## Author Contributions

Takahiro Masuda: Writing – original draft. Ayako Yoshiyama, Sayaka Fujimoto, Yuya Furukawa, Hiroko Jinno, and Soichiro Yoshimura: Resources. Masatsugu Yamamoto and Kimihide Tada: Supervision.

## Consent

The authors declare that written informed consent was obtained for the publication of this manuscript and accompanying images using the consent form provided by the Journal.

## Conflicts of Interest

The authors declare no conflicts of interest.

## Data Availability

The data that support the findings of this study are available on request from the corresponding author. The data are not publicly available due to privacy or ethical restrictions.
